# Complete genome sequence of *Bacillus cereus* A01

**DOI:** 10.1128/mra.01241-23

**Published:** 2024-04-30

**Authors:** Jiahao Li, Xiuyun Wu, Jing Meng

**Affiliations:** 1 The State Key Laboratory of Microbial Technology, Shandong University, Jinan, Shandong, China; 2 Jinan Maternity and Child Care Hospital, Jinan, Shandong, China; DOE Joint Genome Institute, Berkeley, California, USA

**Keywords:** genome

## Abstract

*Bacillus cereus,* a class of facultative aerobic gram-positive bacteria, is frequently isolated from soil, growing plants, and the intestinal tract of insects and mammals. Here, we report the complete genome sequence of *B. cereus* A01, whose total genome length is 6,097,808 bp, with a GC content of 34.92%.

## ANNOUNCEMENT

*Bacillus cereus* is a gram-positive, facultative anaerobic bacterium, which is widely found in soil, water, air, and animal intestines (1). *B. cereus* has abundant secondary metabolites and is one of the commonly used strains of probiotics (2). *B. cereus* A01 was isolated in March 2023 from a stool sample of healthy children in Jinan, Shandong Province, China. After diluting 0.1-g sample with 1-mL 0.9% sterile sodium chloride solution, the mixture was added to yeast peptone medium for enrichment culture and then dropped on blood agar (BA) plates (purchased from QingDao Hopebio-Technology Co., Ltd.) for uniform coating. The plates were incubated in an aerobic incubator at 37°C for 16 h under static conditions. A single colony purified by three rounds of scribing was selected for further identification. The 16S rRNA gene sequence was amplified using PCR with the primer pair 27F and 1492R. The nearly complete 16S rRNA gene was obtained (with the GenBank accession number PP439830) by Sanger dideoxy sequencing. The strain A01 was identified as *B. cereus*. Here, we report the complete genome sequence of *B. cereus* A01.

*B. cereus* A01 was inoculated into yeast peptone medium and was cultured at 37°C for 12 h. Genomic DNA was extracted using the Qiagen Genomic-tip 20/G kit (Qiagen) according to the manufacturer’s instruction. Oxford Nanopore Technologies (ONT) and Illumina libraries were prepared from the same DNA preparation. Specifically, the library for Illumina sequencing was prepared using the TrueLib DNA Library Rapid Prep Kit for Illumina and was sequenced on an Illumina NovaSeq 6000 platform (paired sequencing). The library for ONT sequencing was prepared using the SQK-LSK109 kit and was sequenced on a PromethION48 sequencer with PromethION Flow Cell (R9 Version). Guppy v5.0.16 with high-accuracy mode was used as the base caller (3). All reads were provided as FASTQ files (Illumina, 4,402,246*2 raw pair reads [1,318,148,314 bases, with 99.35% coverage based on reads mapping and raw Q30 of 94.72%]; ONT, 220,372 reads [3,298,622,809 bases, with 98.07% coverage based on reads mapping and the largest, average length, and reads N50 value of 395,702, 14,968.43, and 13,049 bp]). The raw data generated by Illumina sequencing were clipped using fastp v0.23.0 to obtain high-quality clean data. The assembly software Unicycler v0.4.8 (4) was used for the ONT genome assembly. During the assembly process, Pilon v1.22 software (5) was used to map Illumina short sequences to the assembled genome for correction. Genome annotation was performed using the NCBI Prokaryotic Genome Annotation Pipeline (PGAP v5.3) (6). The annotation method was best-placed reference protein set and GeneMarkS-2+. All tools were run with default parameters unless otherwise specified.

The *B. cereus* A01 genome was assembled into two circular replicons, with one circular chromosome (5,507,140 bp) and one circular plasmid (590,668 bp). The total genome length is 6,097,808 bp, containing 5,809 protein-coding genes, 106 tRNAs, 42 rRNAs, and an average GC content (Count (G + C)/Count (A + T + G + C) * 100%) of 34.92%. The whole genome sequence of *B. cereus* A01 is of great value for further functional genome study of *Bacillus*.

## Data Availability

The genome sequence of *B. cereus* A01 is available in GenBank under accession numbers CP139214 and CP139215. The raw sequences are available under Sequence Read Archive (SRA) accession numbers SRR27324557 (Illumina) and SRR27324556 (ONT).
